# Heavy alcohol drinking and subclinical echocardiographic abnormalities of structure and function

**DOI:** 10.1136/openhrt-2020-001457

**Published:** 2021-06-02

**Authors:** Olena Iakunchykova, Henrik Schirmer, Darryl Leong, Sofia Malyutina, Andrew Ryabikov, Maria Averina, Alexander Kudryavtsev, Mikhail Kornev, Ekaterina Voronina, Andrey Paramonov, Tom Wilsgaard, David Leon

**Affiliations:** 1 Department of Community Medicine, UiT The Arctic University of Norway, Tromsø, Norway; 2 Department of Cardiology, Akershus University Hospital, Lorenskog, Norway; 3 Institute of Clinical Medicine, Campus Ahus, University of Oslo, Oslo, Norway; 4 Department of Clinical Medicine, UiT The Arctic University of Norway, Tromsø, Norway; 5 Population Health Research Institute, McMaster University, Hamilton, Ontario, Canada; 6 Branch of Institute of Cytology and Genetics, Siberian Branch of the Russian Academy of Sciences, Research Institute of Internal and Preventive Medicine, Novosibirsk, Russian Federation; 7 Novosibirsk State Medical University, Novosibirsk, Russian Federation; 8 Department of Laboratory Medicine, University Hospital of North Norway, Tromsø, Norway; 9 Department of Innovative Programs, Northern State Medical University, Arkhangelsk, Russia; 10 Central Scientific Research Laboratory, Northern State Medical University, Arkhangelsk, Russian Federation; 11 Department of Non-Communicable Disease Epidemiology, London School of Hygiene & Tropical Medicine, London, UK; 12 International Laboratory For Population and Health, National Research University Higher School of Economics, Moscow, Russia

**Keywords:** heart failure, echocardiography, atrial fibrillation, epidemiology

## Abstract

**Objective:**

The aim of the study is to assess changes in heart structure and function associated with heavy alcohol use by comparing echocardiographic indices in a population-based sample to those in patients admitted to an inpatient facility with severe alcohol problems.

**Methods and results:**

We used data from the Know Your Heart study (2015–2017) which is a cross-sectional study that recruited 2479 participants aged 35–69 years from the general population of the city of Arkhangelsk in Northwest Russia and 278 patients from the Arkhangelsk Regional Psychiatric Hospital with a primary diagnosis related to chronic alcohol use (narcology clinic subsample). The drinking patterns of the population-based sample were characterised in detail. We used regression models controlling for age, sex, smoking, education and waist to hip ratio to evaluate the differences in echocardiographic indices in participants with different drinking patterns. The means of left ventricular end-diastolic diameter and indexed left atrial systolic diameter were increased among heavy drinkers (narcology clinic subsample), while mean left ventricular ejection fraction was decreased in this group compared with the population-based sample. In contrast, the harmful and hazardous drinkers in the population-based sample did not differ from non-problem drinkers with respect to echocardiographic indices of systolic and diastolic function.

**Conclusions:**

Extremely heavy drinking is associated with a specific set of structural and functional abnormalities of the heart that may be regarded as precursors of alcohol-related dilated cardiomyopathy.

**Key messages**

**What is already known about this subject?**

- In some individuals, long-term heavy alcohol consumption results in clinically diagnosed alcoholic cardiomyopathy.

**What does this study add?**

- This study characterised preclinical changes in heart structure and function in high-risk alcohol users using echocardiography.
- Two potential mechanisms are plausible based on observed changes: alcohol-related dilated cardiomyopathy by toxic damage of the heart muscle that could, among other effects, predispose to atrial fibrillation and consequently stroke or heart failure.
- Our study provides insight into the underlying mechanisms that may explain the observed associations between heavy drinking and cardiovascular mortality.

**How might this impact on clinical practice?**

- General practitioners and cardiologists should be more aware of alcohol as a risk factor for cardiovascular disease.
- Screening for alcohol abuse should be done even in patients without symptoms of heart failure.

## Introduction

Long-term heavy alcohol consumption is known to have a direct toxic effect on the heart that can ultimately result in clinically diagnosed alcoholic cardiomyopathy.^1^ However, there will be a continuum of changes in cardiac structure and function in response to heavy drinking which may or may not ultimately result in a diagnosis of cardiomyopathy per se. There have been very few studies of the possible spectrum of such pathophysiological changes despite the fact that they may in themselves increase the risk of serious cardiovascular events.

The most informative study to date was of nearly 50 000 Koreans who attended routine clinical health checks and had alcohol consumption recorded. In this study, heavy drinking (>60 g/day alcohol) was associated with increased odds of left ventricular enlargement, relative wall thickness changes and impaired left ventricular diastolic function (based on septal e′ velocity and E/e′ ratio).^2^ A small Serbian study found no difference in left ventricular ejection fraction in 89 asymptomatic alcoholic patients compared with 30 participants with no alcohol problems.^3^

Most other population-based studies that have investigated echocardiographic characteristics of drinkers have been limited to participants who drank moderately (ranging from abstinence to drinking more 14 standard drinks a week). These have not found any consistent association of alcohol drinking with abnormalities of left ventricular structure and function.^3–10^ The Framingham Heart Study found some evidence that alcohol drinking could affect risk of atrial fibrillation via its impact on left atrial structure.^11^

Most previous studies measured exposure to alcohol in terms of average volume of ethanol consumed in a defined period (a month or a year). However, it may be the case that certain patterns of drinking (eg, episodic binges) are particularly harmful to the heart.^1^ Excessive alcohol consumption, especially characterised by heavy episodic drinking of spirits,^12 13^ is thought to contribute substantially to cardiovascular diseases (CVD) mortality in countries of Eastern Europe.^14 15^ It is hypothesised that in this context alcohol may contribute to CVD mortality through non-ischaemic pathways. This is consistent with previous results we have reported that found elevated levels of N-terminal pro-b-type natriuretic peptide (NT-proBNP) among harmful drinkers in Russia and in Northern Ireland.^16 17^ However, we are not aware of any studies that have looked at the relationship between pattern and volume of heavy alcohol consumption and subclinical abnormalities of cardiac structure.

In this paper, we address many of the limitations of previous work by studying echocardiographic indices of heart structure and function in a sample of patients admitted to a clinical facility with a primary diagnosis of severe alcohol problems in comparison with a population-based sample of adults from the Russian city of Arkhangelsk with well-characterised drinking patterns.

## Methods

### Study design

As part of the Know Your Heart (KYH) study, we recruited 2479 participants from the general population of the city of Arkhangelsk in Northwest Russia in the years 2015–2017. A detailed account of the rationale and description of the methodology of KYH has been published previously.^18^ At the same time, we recruited 278 patients from the Arkhangelsk Regional Psychiatric Hospital with a primary diagnosis related to chronic alcohol drinking.^18^ The latter group is referred to subsequently as the narcology clinic subsample consistent with Russian terminology.

### Study participants

The general population sample from the city Arkhangelsk was recruited at random (stratified by age, sex and district of residence) using the regional health insurance fund register as the sampling frame. Trained interviewers visited the addresses of the randomly selected residents and invited persons of the target age and sex at each address to take part in the study. When successful, an interview was conducted about the health and behaviours of the participant. The response rate was 68% of the addresses where contact with a person of the target age and sex was established and 96% of those interviewed took part in a subsequent health check at the polyclinic of the Northern State Medical University, Arkhangelsk.^18^ As described elsewhere,^16^ a sample of heavy drinkers (narcology clinic subsample) was also recruited from the regional psychiatric hospital where they were undergoing detoxification as inpatients. A total of 278 patients were recruited out of 322 patients invited (85.4%).

In this study, we analysed data on 2345 participants from the general population in Arkhangelsk and 268 persons from the narcology clinic subsample who completed an echocardiographic examination during the health check.

### Data and sample collection

The baseline interview was administered by a trained interviewer using a tablet computer-assisted personal interviewing device. For the general population sample, the interview was done in people’s homes. For the narcology clinic subsample, it was done at the hospital by the same set of trained interviewers. Information was collected on medical history and socioeconomic circumstances, education and lifestyle.

The subsequent health check at the university polyclinic comprised a physical examination (including blood pressure, height, waist and hip circumference, and weight), blood sample collection and echocardiography examination. All participants had a second interview at this stage that recorded medical history, use of medications, alcohol use and smoking.

### Echocardiographic examination

Six trained specialists certified in ultrasound performed the transthoracic echocardiography (ECHO) in the left lateral decubitus position using a 1.5–3.6 MHz phased array sector transducer (Vivid q, GE Healthcare). ECHO was performed according to standardised protocol consisting of B-mode, M-mode as well as pulsed-wave, continuous-wave and colour Doppler assessments in the parasternal view and apical 4-chamber and 2-chamber views. If necessary, breath-hold was used to ensure image quality. Offline reading was performed using EchoPAC (v.113, GE-Vingmed AS, Horten, Norway) by three ECHO specialists (SM, AR and EV) who were blinded to clinical data on each participant. We assessed the reproducibility of offline measurements in 60 participants randomly selected at the initial analytical stage.

### Outcome variables

Left ventricular (LV) and left atrial (LA) volumes were measured from the apical 2-chamber and 4-chamber views using the biplane Simpson’s technique and LV ejection fraction (LVEF) calculated.^19^ LV mass was estimated from M-mode measurements according to current echocardiographic guidelines.^19^ Chamber volumes and dimensions were indexed to body surface area,^19^ LV mass was indexed by height^2.7^.^20^

Doppler measurements of aortic, mitral, pulmonary and tricuspid valve flow were obtained according to current guidelines.^21^ Peak early diastolic mitral annulus velocity (e′) was calculated as the average of peak velocities measured at the septal and lateral annulus by pulsed wave tissue Doppler imaging (TDI), E/e′ ratio was calculated as the ratio of the mitral peak early diastolic filling velocity to mitral annular early diastolic velocity.^21^

Left ventricular hypertrophy (LVH) was defined by LV mass >50 g/m^2.7^ among men and >47 g/m^2.7^ among women.^22^ Systolic dysfunction was defined by LVEF <50%,^22^ increased indexed LV end-diastolic diameter was defined^19^ >3.1 cm/m^2^ among men and >3.2 cm/m^2^ among women, and increased indexed LV end-diastolic volume^22^ was defined >75 mL/m^2^. Enlarged indexed LA volume^21^ was defined >34 mL/m^2^, enlarged indexed LA diameter^19^ >2.3 cm/m^2^.

Diastolic dysfunction was defined based on reference values for tricuspid regurgitation velocity (>2.8 m/s), peak early diastolic mitral annulus velocity e′ (septal <7 cm/s or lateral <10 cm/s), E/e′ ratio (≥14), left atrial volume index (>34 mL/m^2^).^21^

### Exposure variables

We defined two categorical exposure variables. The first was binary and divided the study group into those from the narcology clinic subsample and those from the general population sample. The second exposure variable further categorised the general population sample by drinking pattern of harmful, hazardous, non-problem drinkers, non-drinkers (never drinkers and ex-drinkers) based on self-report of various dimensions of alcohol consumption (online supplemental table 1, online supplemental figure 1).^16^ The choice of drinking patterns as an exposure variable rather than volume of alcohol consumption is justified because very heavy drinkers tend to under-report the amount of consumed alcohol.^23^

10.1136/openhrt-2020-001457.supp1 Supplementary data

### Covariates

Information was available on classical CVD risk factors, including those that are on the potential causal pathway between alcohol use and heart structure and function abnormalities. We adjusted for age, sex, smoking and education as potential confounders. Education was classified into four categories: incomplete secondary or lower; secondary or professional school (general secondary school and/or secondary technical school); incomplete higher or specialised secondary (college or university of up to 3 years); higher (university, 4–6 years). Smoking status was categorised as current smokers, ex-smokers and never-smokers. For current smokers, the number of cigarettes smoked was specified as 1–10/day, 11–20/day, >20/day. Body mass index (BMI) was calculated as weight (kg) divided by height (m) squared. Waist to hip ratio (WHR) was the mean of two measurements of waist divided by the mean of two measurements of hip.

Potential mediators of alcohol use on heart structure and function included use of antihypertensive medications and systolic blood pressure (SBP) and diastolic blood pressure (DBP) (mean of the second and third measurements) because blood pressure is known to increase as a consequence of alcohol use.^24^ We adjusted for this set of mediators separately to evaluate the mediating role of blood pressure distinct from other possible mediators. Use of antihypertensives was determined based on medications reported by the participants if they could be classified as diuretics, beta-blocking agents, calcium channel blockers and agents operating on the renin-angiotensin system.

NT-proBNP was measured using a high sensitivity electrochemiluminescence immunoassay (Roche Diagnostics GmbH, Hitachi, Japan) on a Cobas e411 analyser.

### Statistical methods

To assess the association between patterns of alcohol use and cardiac structure and function, we compared the echocardiographic measurements of the narcology clinic with the general population sample. Next, we compared echocardiographic indices across the categories of alcohol consumption: (1) narcology clinic subsample; (2) general population sample, harmful drinking pattern; (3) general population sample, hazardous drinking pattern; (4) general population sample, non-problem drinkers; (5) general population sample, never drinkers; (6) general population sample, ex-drinkers. The means and proportions were calculated along with 95% CI adjusted for age, sex, operator, reader as least-square means (predicted population margins) computed using general linear regression or logistic regression models.

Multivariable adjusted general linear regression models were used to compare the means of echocardiographic indices across categories of alcohol consumption: model 1 adjusted for age and sex; model 2 adjusted for potential confounders (age, sex, smoking, education, WHR). Model 3 additionally included possible mediators (SBP and DBP, use of blood pressure lowering medications). Ultrasound operators and readers of the echocardiographic images were included into all models as fixed effects to control for potential systematic differences between them. Model assumptions were assessed by visual inspection of residual plots.

Statistical analysis was performed using SAS software V.9.4 (SAS Institute).

## Results

The participants from the narcology clinic were more often men than in the general population sample (76.8% vs 41.7%) and had lower mean age (48.5 vs 53.7 years). Table 1 shows the means and percentages for CVD risk factors in the narcology clinic subsample and general population sample (age-adjusted and sex-adjusted). The participants from the narcology clinic were more likely to be smokers and to be less educated. However, BMI, total and low density lipoproteincholesterol, SBP and use of blood pressure lowering medications were higher among the general population.

**Table 1:** Main cardiovascular risk factors in narcology clinic subsample and the general population sample

		Narcology clinic, N=268	General population sample, N=2345	P value*
% (N) men		76.8 (208)	41.7 (981)	<0.001
Age (years), mean (SD, min, max)		48.5 (8.5, 35.1, 68.6)	53.7 (9.6, 35.3, 69.9)	<0.001
	**Missing N**	Means or percentages* (95% CI)	Means or percentages* (95% CI)	
Current drinkers†	0	100%	91.0% (89.0 to 92.0)	
Current smoking	2	76.3% (69.9 to 81.8)	23.0% (21.3 to 24.9)	<0.001
AUDIT score>=8	0	96.8% (93.2 to 98.5)	7.8% (6.5 to 9.3)	<0.001
CAGE score>=2	0	90.1% (84.9 to 93.6)	11.1% (9.7 to 12.5)	<0.001
Use of antihypertensive medication	0	21.0% (15.8 to 27.3)	35.4% (33.2 to 37.7)	<0.001
BMI (kg/m^2^), mean	0	24.9 (24.2 to 25.7)	27.8 (27.4 to 28.2)	<0.001
Waist to hip ratio, mean	0	0.88 (0.87 to 0.89)	0.88 (0.88 to 0.89)	0.292
Systolic blood pressure (mm Hg), mean	10	127.4 (124.7 to 130.1)	132.8 (131.5 to 134.1)	<0.001
Diastolic blood pressure (mm Hg), mean	10	83.2 (81.5 to 84.8)	83.8 (83.0 to 84.6)	0.419
Ln-transformed NT-proBNP	19	4.95 (4.81 to 5.09)	4.42 (4.35 to 4.49)	<0.001
NT-proBNP >125 pg/mL	19	55.99 (49.27 to 62.5)	28.35 (26.34 to 30.44)	<0.001
Total cholesterol (mmol/L), mean	10	4.99 (4.83 to 5.15)	5.35 (5.27 to 5.42)	<0.001
Low density lipoprotein cholesterol (mmol/L), mean	10	3.23 (3.1 to 3.36)	3.58 (3.52 to 3.65)	<0.001
High density lipoprotein cholesterol (mmol/L), mean	10	1.46 (1.41 to 1.52)	1.44 (1.41 to 1.46)	0.283
No higher education	0	52.8% (46.6 to 59.0)	23.6% (21.9 to 25.4)	<0.001

*Adjusted for age, sex.

†All participants from narcology clinic sample are current drinkers, but were not drinking during the period of admission to narcology clinic.

AUDIT, Alcohol Use Disorders Identification Test; BMI, body mass index; CAGE, Cut down, Annoyed, Guilty, Eye Opener; NT-proBNP, N-terminal pro-b-type natriuretic peptide.

Table 2 presents age-adjusted and sex-adjusted mean values of echocardiographic parameters. Some echocardiographic measurements were not technically possible in some participants, which resulted in different analytic samples for each outcome measure (from 2370 to 2609 participants) (table 2). After controlling for potential confounders, mean LV end-diastolic diameter and mean LA systolic diameter were larger in narcology clinic subsample compared with general population sample, but there was no difference in mean LV mass (table 3). While mean LV end-diastolic volume and mean LA volume were larger in narcology clinic subsample compared with general population sample, differences were not statistically significant after adjustment for confounders (table 3). Among functional measurements, mean LV ejection fraction in the narcology clinic subsample was lower than the general population sample, while mean peak early diastolic mitral annulus velocity (e′) and mean E/e′ ratio were the same as in the general population sample. Additional adjustment for SBP, DBP, and blood pressure lowering medication use as possible mediators did not substantially affect these findings (table 3). The same analysis stratified by sex has given similar results (online supplemental table 2). We did not find any differences in echocardiographic indices between harmful and hazardous drinkers in the general population and non-problem drinkers (online supplemental tables 3-4).

**Table 2:** Echocardiographic indices in narcology clinic subsample and the general population sample

Outcome variables	Missing N	Narcology clinic, N=268	General population sample, N=2345	P value*
		Means* (95% CI)	Means* (95% CI)	
Left ventricular mass/h^2.7^, g/m^2.7^	16	45.58 (43.92 to 47.25)	45.31 (44.51 to 46.11)	0.728
Left ventricular end-diastolic volume biplane/bsa, mL/m^2^	255	42.65 (41.13 to 44.16)	41.6 (40.84 to 42.36)	0.134
Left ventricular end-diastolic diameter/bsa, cm/m^2^	16	2.87 (2.83 to 2.91)	2.75 (2.73 to 2.77)	<0.001
Left atrial systolic diameter/bsa, cm/m^2^	30	2.11 (2.08 to 2.14)	2.01 (2.00 to 2.03)	<0.001
Left ventricular ejection fraction biplane, %	255	54.04 (53.15 to 54.94)	55.94 (55.49 to 56.39)	<0.001
Left atrial volume/bsa, mL/m^2^	52	25.88 (24.86 to 26.9)	25.56 (25.07 to 26.06)	0.506
Peak early diastolic mitral annulus velocity (average e′), (cm/s)	99	10.20 (9.88 to 10.53)	10.54 (10.38 to 10.70)	0.026
E/e′	138	6.84 (6.53 to 7.16)	6.97 (6.82 to 7.13)	0.383

*Adjusted for age, sex, operator, reader.

bsa, body surface area.

**Table 3:** Differences in echocardiographic indices between narcology clinic subsample compared with general population sample

Outcome variables	Model 1* β (95% CI)†	Model 2* β (95% CI)	Model 3* β (95% CI)
Left ventricular mass/h^2.7^, g/m^2.7^	0.27 (−1.27 to 1.81)	−0.25 (−1.81 to 1.31)	0.79 (−0.74 to 2.32)
Left ventricular end-diastolic volume biplane/bsa, mL/m^2^	1.04 (−0.32 to 2.41)	1.28 (−0.21 to 2.77)	1.83 (0.34 to 3.31)
Left ventricular end-diastolic diameter/bsa, cm/m^2^	0.12 (0.08 to 0.16)	0.07 (0.03 to 0.11)	0.07 (0.04 to 0.11)
Left atrial systolic diameter/bsa, cm/m^2^	0.09 (0.07 to 0.12)	0.07 (0.04 to 0.11)	0.08 (0.05 to 0.11)
Left ventricular ejection fraction biplane, %	−1.90 (−2.70 to –1.09)	−1.58 (−2.44 to 0.72)	−1.56 (−2.43 to 0.7)
Left atrial volume/bsa, mL/m^2^	0.32 (−0.62 to 1.26)	0.60 (−0.42 to 1.62)	1.12 (0.10 to 2.14)
Peak early diastolic mitral annulus velocity (average e′) (cm/s)	−0.34 (−0.64 to –0.04)	−0.11 (−0.42 to 0.2)	−0.15 (−0.45 to 0.15)
E/e′	−0.13 (−0.42 to 0.16)	−0.14 (−0.45 to 0.18)	0.05 (−0.26 to 0.36)

*Model 1: Adjusted for age and sex. Model 2: Additionally adjusted for education and smoking, WHR. Model 3: Additionally adjusted for SBP, DBP, blood pressure medication. All models are adjusted for operator and reader.

†β, linear regression coefficient for the difference between narcology clinic subsample and general population sample (positive value indicates higher mean in the narcology clinic subsample).

bsa, body surface area; DBP, diastolic blood pressure; SBP, systolic blood pressure; WHR, waist to hip ratio.

We also examined differences in echocardiographic parameters between the general population sample and the narcology clinic subsample by comparing the prevalence of dysfunction defined using normal value thresholds. As shown in online supplemental table 5, these same patterns of association appeared as seen for the comparison of means. The general population sample prevalence of increased LV end-diastolic volume was 0.2% (5), of increased LV end-diastolic diameter 5.4% (126), of LVH 33.8% (790), while in the narcology clinic subsample, the prevalence of those were 0.8% (2), 9.0% (24) and 19.8% (53), respectively. Systolic dysfunction (LV ejection fraction <50%) was present in 10.2% (215) of the general population sample and 14.6% (37) of the narcology clinic subsample. Enlarged LA volume was present in 12.1% (279) of the general population sample and 15.0% (40) of the narcology clinic subsample, increased LA diameter was present in 9.9% (230) of the general population, compared with 13.5% (36) of the narcology clinic subsample. There were 7 (2.7%) participants with probable and 1 (0.4%) with definite diastolic dysfunction in the narcology clinic subsample; and 80 (3.6%) with probable and 12 (0.6%) with definite diastolic dysfunction in the general population sample.

NT-proBNP was elevated above 125 pg/mL among 41.35% (110) of the narcology clinic subsample participants compared with 32.61% (763) of the general population sample. The mean levels of ln-transformed NT-proBNP controlled for age and sex were higher in the narcology clinic subsample compared with the general population sample (table 1). Finally, we examined the relationship between echocardiographic indices and NT-proBNP (online supplemental table 6). As expected, moderate correlations were found between echocardiographic markers of cardiac remodelling and function, and NT-proBNP. The correlation was strongest for LV mass, LV end-diastolic diameter, LA left atrial systolic diameter, LA volume, E/e′.

### Sensitivity analysis

Excluding never drinkers and ex-drinkers from the general population sample did not have any impact on the results (online supplemental table 7).

## Discussion

We found evidence of abnormalities in cardiac structure and function among heavy drinkers from a narcology clinic in Arkhangelsk (Russia) compared with the Arkhangelsk general population. Specifically, the narcology clinic subsample exhibited adverse LV remodelling (larger LV end-diastolic diameter and lower LV ejection fraction), which may indicate early signs of toxic dilated cardiomyopathy. The evidence regarding adverse LA remodelling in the narcology clinic subsample (which may represent the sequelae of systolic and diastolic LV dysfunction) was equivocal: LA diameter was larger, but differences with the general population sample in LA volume were not statistically significant after adjustment for confounders. This may in part reflect the fact that in our study there may have been greater imprecision in the measurement of volume compared with diameter, leading to an artefactual attenuation in the strength of the true association. Diastolic dysfunction as measured by decreased peak early diastolic mitral annular velocity was observed in unadjusted models, but the regression coefficients decreased to zero after adjustment for smoking, education and WHR. Ours is the largest study that has been able to compare various differences in cardiac structure and function among those with a clinical diagnosis of alcohol problems and a sample of the general population stratified by drinking pattern.

In the population-based sample, we found no clear evidence for an association between patterns of alcohol consumption and echocardiographic indices of global left ventricular systolic and diastolic function.

### Previous research

Our finding of increased LV end-diastolic diameter is consistent with the results of a large community-based study of nearly 50 000 participants conducted in Korea.^2^ However, unlike this study where heavy drinking (more than 60 g of alcohol per day) was associated with increased odds of left ventricular hypertrophy and increase in E/e′ compared with abstainers, we did not observe increase in LV mass or E/e′.^2^ Also, we observed decreased LV ejection fraction in very heavy drinkers in our study, while the Korean study did not report findings on LV ejection fraction. Contrary to this, a small study of chronic asymptomatic alcoholics found no differences in LV ejection fraction compared with those without alcohol problems.^3^

Apart from the studies mentioned above, the focus of most research on alcohol in relation to echocardiographic indices was on moderate drinking. It is plausible that it is only chronic heavy episodic drinking that gives rise to the detectable changes in cardiac structure or function. Nevertheless, for completeness, we note the following conclusions from the studies of moderate drinkers. Reduced LV ejection fraction among alcohol drinkers was found in some studies,^4–7^ but not in others.^8–10^ Similarly, the dimensions and mass of the left ventricle were found to be increased among drinkers in some studies^4 8–10^ not in all.^6^ LA dimensions and indices of diastolic dysfunction were increased among drinkers in some studies,^4 9 11^ but not in others.^6 8 10^

### Clinical implications

The adverse LV remodelling we observed in the narcology subsample may represent a subclinical end of the spectrum of dilated cardiomyopathy, which is associated with an increased risk of ventricular arrhythmia. This finding is also supported by elevated NT-proBNP, a marker of cardiac wall stretch, in the narcology clinic subsample compared with the general population sample.^16^ There is little direct evidence for the association between long-term alcohol consumption and ventricular arrhythmias, although it is postulated that adverse LV remodelling may play a role in the observed association between heavy alcohol consumption and sudden cardiac death.^25^ Atrial fibrillation is the most common arrhythmia linked to the alcohol consumption,^26^ and the potential causal link between alcohol use and atrial fibrillation is well known for years and recently supported by evidence from a randomised controlled trial, which showed a reduction of atrial fibrillation recurrences after cessation of alcohol consumption by regular drinkers.^27^

We did not find changes in echocardiographic abnormalities in harmful and hazardous drinkers in the general population sample compared with non-problem drinkers. The number of people in these groups in our study was relatively small and thus we might be underpowered to detect minor subclinical changes in these groups. Moreover, both the harmful and hazardous drinkers in the population-based sample had lower levels of two blood-based biomarkers (gamma-glutamyl transferase and carbohydrate-deficient transferrin) of heavy drinking than the narcology subsample participants (online supplemental table 1). However, NT-proBNP was elevated both in harmful drinkers as well as in the narcology clinic subsample as reported in our previous analysis of the KYH study.^16^

Among the potential mechanisms that are suggested to mediate the harmful action of alcohol on the heart are increased blood pressure and a direct effect of the toxic alcohol metabolites on the heart muscle. Blood pressure has a linear dose–response relationship with volume of alcohol consumed^24^ and is an established risk factor for CVD. Toxic alcohol metabolites affect heart muscle by increasing oxidative stress, triggering cell apoptosis.^28^ In our study, controlling for blood pressure in the regression model did not substantially change the estimates of the association between alcohol use and echocardiographic abnormalities. This suggests that hypertension may not play a large role in mediating the relationship observed between heavy alcohol use and the echocardiographic abnormalities observed.

Furthermore, the most consistent echocardiographic abnormality in our study was left ventricular dilatation, whereas hypertensive heart disease usually manifests as diastolic dysfunction and left ventricular hypertrophy. This, coupled with the lack of a strong mediating effect of hypertension, suggests that the primary abnormality related to heavy alcohol use is cardiomyopathy due to a direct toxic effect. Abnormal systolic function reflected by LV ejection fraction was detected in our study in the narcology clinic subsample, but diastolic function was not impaired. Thus, functional abnormalities are less prominent and may develop at a later pathological stage.

### Limitations

There are inherent limitations to using echocardiography compared with MRI due to higher measurement error for some echocardiographic indices.^29^ Also, estimating right ventricular systolic pressure from tricuspid regurgitation velocity likely led to underestimation of diastolic dysfunction in our study. Therefore, we opted to assess the components of diastolic dysfunction (LA diameter, LA volume, e′ and E/e′) separately as continuous variables. Although we observed an increased LA diameter in the narcology clinic sample compared with the general population sample, differences in LA volume between these two groups were not statistically significant after adjustment for confounders. This discrepancy between two indices of LA size may be explained by high measurement error for biplane technique. The true differences are higher than observed in the studies of outcome variables characterised by high measurement error. Better characterisation of heart function would be possible with novel echocardiography techniques like global longitudinal strain measurement. They may be more sensitive to subtle changes that would not be detected with the conventional echocardiography techniques. We cannot exclude that the results are affected by residual confounding by smoking or malnourishment in the narcology clinic subsample, as well as the effect of medication for detoxification after alcohol withdrawal in this group.

## Conclusions

The characterisation of preclinical changes in heart structure and function in high-risk alcohol users provides insight about the link between alcohol use and cardiovascular mortality. One potential mechanism is alcohol-related dilated cardiomyopathy by toxic damage of the heart muscle. This could predispose to atrial fibrillation and consequently stroke or heart failure. Our results throw further light on some of the underlying mechanisms that may help explain the observed associations between heavy drinking and cardiovascular mortality.

## Data Availability

Data are available on reasonable request. Requests to access the dataset of KYH study from bona fide researchers may be sent to the International Project on Cardiovascular Disease in Russia (https://metadata.Knowyourheart.Science/)
