# Supplementary material for: Heavy alcohol drinking and subclinical echocardiographic abnormalities of structure and function
Source: Open Heart. 2021 Jun 2;8(1):e001457. doi: 10.1136/openhrt-2020-001457 (PMC8174503; doi:10.1136/openhrt-2020-001457)
Supplement: Supplementary data [file openhrt-2020-001457supp001.pdf]

SUPPLEMENTARY MATERIAL

Category of drinker	Drinking alcoholic beverages during last 12 month	AUDIT>=8	CAGE>=2	Harmful Russian drinking pattern [*]
General population sample				
Harmful drinker	✓	✓	✓	✓
Hazardous drinker	✓	✓ for one or two instruments		
Non-problem drinker	✓	✗	✗	✗
Non-drinker	✗	✗	✗	✗

Supplementary Figure 1. The assignment scheme of general population sample into categories by drinking status (1) harmful drinkers (2) hazardous drinkers (3) non-problem drinkers (4) non-drinkers. Reproduced with permission Iakunchykova O, Averina M, Kudryavtsev AV, Wilsgaard T, Soloviev A, Schirmer H, Cook S, Leon DA. Evidence for a direct harmful effect of alcohol on myocardial health: a large cross-sectional study of consumption patterns and cardiovascular disease risk biomarkers from Northwest Russia, 2015 to 2017. Journal of the American Heart Association. 2020 Jan 7;9(1):e014491.Copyright 2020, The Authors. Published on behalf of the American Heart Association, Inc., by Wiley Blackwell.

[*] Twice weekly or more frequency of hangover and/or excessive drunkenness and/or sleeping in clothes at night because of drunkenness and/or failing their family or personal obligations because of drinking and/or drinking non-beverage alcohols (sources of ethanol not intended for drinking such as medicinal tinctures) and/or one or more episodes of zapoi (a period of 2 or more days of being drunk during which a participant is withdrawn from normal social life).

Supplementary Table 1. Descriptive measures of alcohol use by categories of drinking pattern. Reproduced with permission Iakunchykova O, Averina M, Kudryavtsev AV, Wilsgaard T, Soloviev A, Schirmer H, Cook S, Leon DA. Evidence for a direct harmful effect of alcohol on myocardial health: a large cross-sectional study of consumption patterns and cardiovascular disease risk biomarkers from Northwest Russia, 2015 to 2017. Journal of the American Heart Association. 2020 Jan 7;9(1):e014491. Copyright 2020, The Authors. Published on behalf of the American Heart Association, Inc., by Wiley Blackwell.

		General population sample				
	Narcology clinic sample n=271	Harmful drinkers n=71	Hazardous drinkers n=424	Non-problem drinking n= 1632	Non-drinkers [‡] n = 227	P-value [*]
Combined biomarker of heavy alcohol use (GGT and GDT) >=4, N (%)	135 (50.9)	27 (38.6)	55 (14.0)	23 (5.2)	0	<0.001
Have asked for help of narcologist or social worker regarding drinking problem, N (%)	271 (100)	26 (36.6)	27 (6.4)	12 (0.8)	19 (23.2) [d]	<0.001
Drinking more than 40 g of alcohol per day, N (%)[†]	62 (23.7)	26 (36.6)	48 (11.3)	12 (0.7)	0	<0.001
Binge drinking (60 g of alcohol per drinking occasion) at least once a month, N (%)	189 (70.5)	49 (69.0)	215 (51.9)	76 (4.8)	0	<0.001
Alcohol consumed per year (liter), mean [†]	15.0	19.0	8.5	1.9	0	<0.001
Alcohol consumed per day (gram), mean [†]	33.45	40.09	18.41	4.04	0.00	<0.001
GGT (U/L)[‖]	68.02	44.39	38.48	25.03	23.69	<0.001
CDT (%)[‖]	1.64	1.60	0.94	0.74	0.53	<0.001

[*] Test for linear trend, adjusted for age and sex.

[†] Alcohol consumption recorded for the last 12 month

[‡] Non-drinkers include lifetime abstainers and ex-drinkers

[§] Among ex-drinkers

[‖] Geometric mean, GGT, Gamma glutamyl transferase; CDT, Carbohydrate Deficient Transferrin

Supplementary Table 2. Echocardiographic indices in narcology clinic subsample and the general population sample by sex.

	Narcology clinic, N=268	General population sample, N=2345	p-value*
	Means * (95% CI)	Means * (95% CI)	
Men			
left ventricular mass/h^2.7, g/m^2.7	43.92 (41.82, 46.03)	45.22 (43.97, 46.47)	0.168
left ventricular end diastolic volume biplane /bsa , ml/m^2	45.36 (43.19, 47.54)	44.08 (42.77, 45.38)	0.177
left ventricular end diastolic diameter /bsa, cm/m^2	2.85 (2.8, 2.9)	2.7 (2.67, 2.73)	<0.001
left atrial systolic diameter/bsa, cm/m^2	2.07 (2.03, 2.11)	1.97 (1.95, 1.99)	<0.001
left ventricular ejection fraction biplane, %	53.84 (52.6, 55.07)	55.49 (54.75, 56.23)	0.002
left atrial volume /bsa, ml/m^2	25.63 (24.27, 26.99)	25.74 (24.93, 26.56)	0.849
Peak early diastolic mitral annulus velocity (Average e'), (cm/s)	10.14 (9.71, 10.57)	10.16 (9.9, 10.41)	0.934
E/e'	6.23 (5.78, 6.67)	6.57 (6.3, 6.84)	0.077
Women			
left ventricular mass/h^2.7, g/m^2.7	48.27 (45.19, 51.34)	45.28 (44.22, 46.33)	0.047
left ventricular end diastolic volume biplane /bsa , ml/m^2	39.57 (37.19, 41.95)	39.51 (38.62, 40.4)	0.959
left ventricular end diastolic diameter /bsa, cm/m^2	2.84 (2.77, 2.91)	2.79 (2.77, 2.81)	0.146
left atrial systolic diameter/bsa, cm/m^2	2.16 (2.1, 2.21)	2.05 (2.03, 2.07)	<0.001
left ventricular ejection fraction biplane, %	54.24 (52.76, 55.71)	56.38 (55.83, 56.92)	0.003
left atrial volume /bsa, ml/m^2	26.81 (25, 28.62)	25.48 (24.86, 26.11)	0.132
Peak early diastolic mitral annulus velocity (Average e'), (cm/s)	9.8 (9.23, 10.37)	10.88 (10.68, 11.07)	<0.001
E/e'	7.66 (7.13, 8.2)	7.28 (7.09, 7.46)	0.138

CI, confidence interval

*Adjusted for age, operator, reader

Supplementary Table 3. Means of echocardiographic indices by alcohol drinking pattern*

		General population sample				
	Narcology clinic sample n=268	Harmful drinkers n=71	Hazardous drinkers n=422	Non-drinkers (never drinkers) n = 145	Non-drinkers (ex-drinkers) n=106	Non-problem drinkers n= 1618
left ventricular mass/h^2.7, g/m^2.7	45.68 (44.01, 47.35)	44.63 (41.83, 47.43)	46.42 (45.12, 47.72)	44.53 (42.6, 46.46)	44.78 (42.5, 47.07)	45.16 (44.29, 46.03)
left ventricular end diastolic volume biplane /bsa , ml/m^2	42.59 (41.07, 44.11)	41.47 (38.96, 43.98)	41.11 (39.92, 42.3)	41.28 (39.5, 43.06)	41.18 (39.13, 43.23)	41.81 (40.98, 42.63)
left ventricular end diastolic diameter /bsa, cm/m^2	2.87 (2.83, 2.91)	2.78 (2.72, 2.85)	2.75 (2.72, 2.78)	2.74 (2.69, 2.78)	2.82 (2.77, 2.87)	2.74 (2.72, 2.76)
left atrial systolic diameter/bsa, cm/m^2	2.11 (2.08, 2.14)	2.06 (2.01, 2.11)	2.02 (2, 2.04)	2.02 (1.98, 2.05)	2.02 (1.98, 2.06)	2.01 (1.99, 2.02)
left ventricular ejection fraction biplane, %	54.09 (53.2, 54.99)	56.79 (55.31, 58.26)	56.02 (55.32, 56.72)	55.35 (54.3, 56.4)	56.4 (55.19, 57.61)	55.93 (55.44, 56.41)
left atrial volume /bsa, ml/m^2	25.87 (24.85, 26.9)	25.37 (23.67, 27.08)	25.37 (24.57, 26.18)	25.5 (24.31, 26.69)	25.99 (24.6, 27.38)	25.61 (25.07, 26.15)
Peak early diastolic mitral annulus velocity (Average e'), (cm/s)	10.14 (9.82, 10.47)	10.2 (9.66, 10.75)	10.13 (9.87, 10.38)	10.53 (10.15, 10.92)	10.25 (9.81, 10.69)	10.69 (10.51, 10.86)
E/e'	6.86 (6.54, 7.18)	7.12 (6.58, 7.66)	7.07 (6.82, 7.32)	7.33 (6.96, 7.71)	7.31 (6.87, 7.74)	6.88 (6.71, 7.05)

*Adjusted for age, sex, operator, reader

Supplementary Table 4. The differences in echocardiographic indices by drinking pattern*, β (95% CI)†

	Narcology clinic sample n=268	General population sample				
		Harmful drinkers n=71	Hazardous drinkers n=422	Non-drinkers (never drinkers) n = 145	Non-drinkers (ex-drinkers) n=106	Non-problem drinkers n= 1618
left ventricular mass/h^2.7, g/m^2.7	0.52 (-1.09, 2.13)	-0.53 (-3.33, 2.28)	1.26 (-0.02, 2.55)	-0.63 (-2.57, 1.3)	-0.38 (-2.61, 1.86)	0 (Reference)
left ventricular end diastolic volume biplane /bsa , ml/m^2	0.78 (-0.64, 2.21)	-0.34 (-2.84, 2.17)	-0.7 (-1.86, 0.46)	-0.53 (-2.3, 1.25)	-0.62 (-2.61, 1.37)	0 (Reference)
left ventricular end diastolic diameter /bsa, cm/m^2	0.13 (0.09, 0.17)	0.04 (-0.03, 0.1)	0.01 (-0.02, 0.04)	0 (-0.05, 0.04)	0.08 (0.03, 0.13)	0 (Reference)
left atrial systolic diameter/bsa, cm/m^2	0.10 (0.07, 0.13)	0.05 (0, 0.1)	0.01 (-0.01, 0.04)	0.01 (-0.02, 0.05)	0.02 (-0.03, 0.06)	0 (Reference)
left ventricular ejection fraction biplane, %	-1.83 (-2.67, -0.99)	0.86 (-0.62, 2.34)	0.09 (-0.59, 0.78)	-0.58 (-1.62, 0.47)	0.48 (-0.70, 1.65)	0 (Reference)
left atrial volume /bsa, ml/m^2	0.26 (-0.72, 1.24)	-0.24 (-1.94, 1.47)	-0.24 (-1.02, 0.55)	-0.11 (-1.3, 1.08)	0.37 (-0.99, 1.73)	0 (Reference)
Peak early diastolic mitral annulus velocity (Average e'), (cm/s)	-0.54 (-0.85, -0.23)	-0.48 (-1.03, 0.07)	-0.56 (-0.81, -0.31)	-0.15 (-0.53, 0.23)	-0.43 (-0.87, 0)	0 (Reference)
E/e'	-0.02 (-0.32, 0.29)	0.24 (-0.3, 0.79)	0.19 (-0.05, 0.44)	0.45 (0.08, 0.83)	0.43 (0, 0.85)	0 (Reference)

*Adjusted for age, sex, operator, reader

†β (95% CI), Linear regression coefficient for the difference between non-problem drinkers (reference category), other groups in the general population sample, and narcology clinic subsample ; CI, confidence interval

Supplementary Table 5. Prevalence of abnormal values for echocardiographic indices in the narcology clinic subsample and the general population sample

Outcome variables	Missing N	Narcology clinic, N=268 Percent (N)	General population sample, N=2345 Percent (N)	OR (95% CI)* (narcology vs general population)	p-value*
left ventricular mass > 50 g/m^2.7 (men), > 47 g/m^2.7 women	16	19.8% (53)	33.8% (790)	0.77 (0.55, 1.08)	0.133
Left ventricular end diastolic volume biplane /bsa , >75 ml/m^2	255	0.8% (2)	0.2% (5)	2.2 (0.4, 12.21)	0.366
Left ventricular end diastolic diameter /bsa, >3.1 cm/m^2 (men), >3.2 cm/m^2 (women)	16	9.0% (24)	5.4% (126)	2.11 (1.3, 3.43)	0.003
Left atrial systolic diameter/bsa, >2.3 cm/m^2	30	13.5% (36)	9.9% (230)	3.34 (2.18, 5.12)	<0.001
LV systolic dysfunction (LV ejection fraction <50%)	255	14.6% (37)	10.2% (215)	1.53 (1.03, 2.27)	0.036
Left atrial volume/bsa, > 34 ml/m^2	52	15.0% (40)	12.1% (279)	1.45 (1.1, 1.92)	0.009
Peak early diastolic mitral annulus velocity (Average e') <7 cm/s	99	9.5% (25)	11.6% (262)	1.31 (0.82, 2.1)	0.263
E/e' ≥ 14	138	0.8% (2)	1.4% (31)	1.26 (0.28, 5.58)	0.765
Diastolic dysfunction (probable or definite)†	170	3.1% (8)	4.2% (92)	1.58 (0.72, 3.47)	0.252

* Odds ratio (OR) of having an abnormal value for echocardiographic parameter in narcology clinic subsample versus general population sample, adjusted for age and sex, CI – Confidence Interval

† Diastolic dysfunction was defined based on reference values for tricuspid regurgitation velocity (> 2.8 m/s), peak early diastolic mitral annulus velocity e' (septal <7 cm/sec or lateral <10 cm/sec), E/e' ratio (≥14), left atrial volume index (>34 ml/m2).

Supplementary Table 6. Person's correlation coefficient between echocardiographic indices and ln-transformed NT-proBNP

Echocardiographic indices	Person's correlation with ln-transformed NT-proBNP	p-value
left ventricular mass/h^2.7	0.30	<0.001
left ventricular end diastolic volume biplane /bsa	0.03	0.107
left ventricular end diastolic diameter/ bsa	0.27	<0.001
left atrial systolic diameter/bsa	0.37	<0.001
left ventricular ejection fraction biplane	-0.07	<0.001
left atrial volume /bsa	0.32	<0.001
Peak early diastolic mitral annulus velocity (Average e'), (cm/s)	-0.22	<0.001
E/e'	0.32	<0.001

Supplementary Table 7. Differences in echocardiographic indices between narcology clinic subsample and general population sample excluding all non-drinkers (never- and ex-drinkers) from analysis

	Model 1*<br>β (95% CI)†	Model 2*<br>β (95% CI)	Model 3*<br>β (95% CI)
left ventricular mass/h^2.7, g/m^2.7	0.17 (-1.38, 1.73)	-0.26 (-1.85, 1.33)	0.73 (-0.82, 2.29)
left ventricular end diastolic volume biplane /bsa , ml/m^2	0.96 (-0.43, 2.36)	1.13 (-0.40, 2.66)	1.63 (0.10, 3.16)
left ventricular end diastolic diameter /bsa, cm/m^2	0.12 (0.08, 0.15)	0.06 (0.02, 0.10)	0.07 (0.03, 0.10)
left atrial systolic diameter/bsa, cm/m^2	0.09 (0.06, 0.12)	0.07 (0.04, 0.1)	0.08 (0.05, 0.11)
left ventricular ejection fraction biplane, %	-1.93 (-2.75, -1.12)	-1.61 (-2.49, -0.73)	-1.6 (-2.49, -0.72)
left atrial volume /bsa, ml/m^2	0.32 (-0.64, 1.28)	0.65 (-0.40, 1.69)	1.16 (0.12, 2.21)
Peak early diastolic mitral annulus velocity (Average e'), (cm/s)	-0.35 (-0.65, -0.05)	-0.15 (-0.46, 0.16)	-0.20 (-0.50, 0.10)
E/e'	-0.08 (-0.36, 0.20)	-0.10 (-0.40, 0.20)	0.09 (-0.20, 0.39)

*Model 1: adjusted for age and sex; Model 2: additionally adjusted for education and smoking, WHR; Model 3: additionally adjusted for SBP, DBP, blood pressure medication. All models are adjusted for operator and reader.

†β, Linear regression coefficient for the difference between narcology clinic subsample and general population sample (positive value indicates higher mean in the narcology clinic subsample); CI, confidence interval
